# Social Psychological Origins of Conspiracy Theories: The Case of the Jewish Conspiracy Theory in Malaysia

**DOI:** 10.3389/fpsyg.2012.00280

**Published:** 2012-08-06

**Authors:** Viren Swami

**Affiliations:** ^1^Department of Psychology, University of WestminsterLondon, UK; ^2^Department of Psychology, HELP University CollegeKuala Lumpur, Malaysia

**Keywords:** conspiracy theories, anti-semitism, modern racism, monological belief system, Malaysia

## Abstract

Two studies examined correlates of belief in a Jewish conspiracy theory among Malays in Malaysia, a culture in which state-directed conspiracism as a means of dealing with perceived external and internal threats is widespread. In Study 1, 368 participants from Kuala Lumpur, Malaysia, completed a novel measure of belief in a Jewish conspiracy theory, along with measures of general conspiracist ideation, and anomie. Initial analysis showed that the novel scale factorially reduced to a single dimension. Further analysis showed that belief in the Jewish conspiracy theory was only significantly associated with general conspiracist ideation, but the strength of the association was weak. In Study 2, 314 participants completed the measure of belief in the Jewish conspiracy theory, along with measures of general conspiracist ideation, and ideological attitudes. Results showed that belief in the Jewish conspiracy theory was associated with anti-Israeli attitudes, modern racism directed at the Chinese, right-wing authoritarianism, and social dominance orientation. General conspiracist ideation did not emerge as a significant predictor once other variables had been accounted for. These results suggest that there may be specific cultural and social psychological forces that drive belief in the Jewish conspiracy theory within the Malaysian context. Specifically, belief in the Jewish conspiracy theory among Malaysian Malays appears to serve ideological needs and as a mask for anti-Chinese sentiment, which may in turn reaffirm their perceived ability to shape socio-political processes.

## Social Psychological Origins of Conspiracy Theories: The Case of the Jewish Conspiracy Theory in Malaysia

In recent years, scholars have paid increasing attention to beliefs in false narratives that exaggerate, idealize, or misconstrue reality, such as beliefs in myths of science (Swami et al., 2012b) and pseudo-history (Allchin, 2004). A related aspect of this literature concerns belief in conspiracy theories, defined as a subset of false narratives in which the ultimate cause of an event is believed to be due to a malevolent plot by multiple actors working together (Goldberg, 2001; Barkun, 2003; Bale, 2007; Swami and Furnham, 2012a). Although this definition of a conspiracy theory is not exhaustive, it does capture the crux of most such beliefs, including the suggestion that the Central Intelligence Agency (CIA) were responsible for the assassination of John F. Kennedy and that the September 11, 2001, terrorist attacks were allowed to happen by high-ranking officials in the United States government.

The study of conspiracy theories deserves greater coverage for a number of reasons. First, they appear to be relatively widespread: since the early 1990s, for example, polls have consistently indicated that upwards of 70% of Americans believe some form of conspiracy was responsible for President Kennedy’s death (Goertzel, 1994; McHoskey, 1995; Southwell and Twist, 2004). Second, the outcomes of belief in conspiracy theories for civic society is, at best, currently uncertain. On the one hand, some scholars believe that conspiracy theories should be viewed as cultural practices that afford citizens opportunities to address the credibility of governance (Sasson, 1995; Fenster, 1999; Hellinger, 2003) or as attempts by lay individuals to create “naïve deconstructionist history” (Rudmin, 2003). On the other hand, because the critique of power offered by conspiracy theories is often simplistic, they are susceptible to racist and exclusionary narratives, which in turn create discord and public mistrust (Fenster, 1999; Miller, 2002; Räikkä, 2008).

In this light, it is perhaps unsurprising that scholars have called for increased attention into the psychology of conspiracy theories (Swami and Coles, 2010), particularly the functions served by these narratives. Until relatively recently, much of the extant literature on conspiracy theories had been dominated by Hofstadter’s (1966) seminal discussion of “paranoid styles” in American politics, in which he attempted to describe “political personalities” that subscribed to right-wing conspiracy theories in clinical terms. Influenced by Hofstadter, many scholars came to view conspiracy theorists as paranoid and delusional; that is, conspiracy theories were viewed as the products of extreme paranoia, delusional thinking, and narcissism (see Robins and Post, 1997). This individual or collective pathology was thought to effectively stunt any form of socio-political action by conspiracy theorists (Groh, 1987), which in turn heightened their paranoia.

Recent work has provided some support for the idea that conspiracy theorists have particular personality profiles marked by paranoia and delusional thinking. For example, two studies have reported that belief in conspiracy theories is positively associated with schizotypal tendencies, that is, the tendency to be suspicious, paranoid, and experience magical thinking and unusual beliefs (Darwin et al., 2011; Swami et al., submitted). In addition, belief in conspiracy theories has also been found to correlate positively with paranormal beliefs (Darwin et al., 2011; Swami et al., 2011), that is beliefs in processes for which there is currently little supportive empirical evidence. Even so, the lens of psychopathology has been critiqued as being reductionist and purely descriptive, as it fails to consider the influence of political structures on conspiracist ideation (Gray, 2008; see also Sunstein and Vermeule, 2009).

Rather, recent work on conspiracy theories has borrowed from Marx’s theory of alienation (see Parry, 1969) to argue that such narratives emerge at times when individuals feel powerless, disadvantaged, or voiceless (Waters, 1997; Fenster, 1999; Pratt, 2003; Whitson and Galinsky, 2008). More specifically, it is argued that some phenomena, such as terrorist attacks, are difficult to comprehend, emotive, lack reliable explanatory frameworks, and remove individual agency (Kravitz, 1999; Parker, 2001; Swami and Furnham, 2012a). In such cases, conspiracy theories help to simplify those phenomena and offer a coherent explanation that may not otherwise be forthcoming. Moreover, by personifying the agents of a conspiracy, conspiracist narratives may allow individuals to reaffirm their ability to shape the socio-political process. In so doing, conspiracy theories serve to ameliorate feelings of powerlessness and fill a need for certainty and control (Miller, 2002; Pratt, 2003; Leman, 2007; Swami and Coles, 2010; Swami and Furnham, 2012a).

Based on this perspective, a number of recent studies have focused on individual difference factors that make belief in conspiracy theories more likely. For example, there is evidence that conspiracist ideation is associated with a range of individual difference traits that signify individual and political anomie. Thus, studies have reported significant associations between belief in conspiracy theories and low self-esteem, feelings of powerlessness, distrust in authority, and political cynicism (Goertzel, 1994; Abalakina-Paap et al., 1999; Swami et al., 2010, 2011). In this view, the alienation felt or experienced by some members of society lead them to adopt conspiracy theories as a means of explaining and understanding their relative powerlessness while simultaneously affording them a means of addressing that imbalance (Swami and Furnham, 2012a).

Although anomie figures prominently in the extant literature on conspiracy theories, it is by no means the only explanation. Goertzel (1994) has suggested that conspiracy theories form part of a “monological belief system,” such that the adoption of a conspiracist worldview makes the assimilation of novel conspiracy theories more likely. In support of this perspective, recent studies have indicated that a conspiracist worldview is associated with stronger belief in specific and newly emerging conspiracy theories (Swami et al., 2010, submitted; Swami and Furnham, 2012b), as well as fictional conspiracy theories (Swami et al., 2011), even if such narratives contain internal contradictions (Wood et al., in press). According to Goertzel (1994), different conspiracy theories support each other and, through their assimilation, a conspiracist explanation is preferred for any event requiring explanation.

Although this body of work continues to grow, an important limitation is that the associations between conspiracist ideation and individual difference variables have only been studied among Western populations. This is an important oversight because, although conspiracy theories exist in many cultures worldwide (Groh, 1987; Zonis and Joseph, 1994; Gentzkow and Shapiro, 2004), the extant literature has not examined antecedents of conspiracist ideation among non-Western populations. It is possible that conspiracy theories fulfill different roles as a function of local identity, culture, or the geopolitical context (Ashraf, 1997; Gray, 2008). For example, variations in the way in which different groups interpret and understand the same event (e.g., the denial of the negative impacts of slavery in the United States; Milburn and Conrad, 1996) suggest that local identities are important in terms of understanding conspiracy theories (Knight, 2000).

To overcome the dearth of research on conspiracy theories among non-Western populations, the present study sought to examine antecedents of the Jewish conspiracy theory in Malaysia. This particular conspiracy theory is far-ranging but generally borrows from early European anti-Semitism and posits a Jewish conspiracy for world domination (Johnson, 1987; Glaeser, 2005). In Malaysia, although this conspiracy was not created *de novo* (Reid, 2010), it has been fueled in particular by a sense of Muslim victimhood and defeat (Suciu, 2008). Furthermore, although the content of this conspiracy theory shares similarities with Middle Eastern and early European anti-Semitism (Schulze, 2006), there are a number of context-specific factors that help explain the prominence of the conspiracy theory in Malaysia (and neighboring Indonesia; Siegel, 2000; Burhanuddin, 2007). First is the absence of an established Jewish population in the region, which has given rise to an imagined or “perceived Jew” (as opposed to the “real Jew” depicted as living in Israel or among the Diaspora), whose influence takes various forms. More specifically, it has been suggested that anti-Jewish conspiracy theorizing in Malaysia and Indonesia reflects displaced resentment of minority Chinese (Siegel, 2000; Hadler, 2004; Burhanuddin, 2007): because government policy ostensibly forbids the expression of anti-Chinese sentiment, resentment of the Chinese minority may have found a voice through anti-Semitism directed at Jews.

Second, and unlike Western Europe and the United States where Jewish conspiracy theories tend to be limited to extremist groups (Billig, 1978, 1988, 1989; Fielding, 1981; Copsey, 2004), the conspiracist narrative about Jews is both widespread and public in Malaysia. Indeed, one characteristic of Malaysian conspiracism is the role of the state in narrating and perpetuating conspiracy theories (Reid, 2010). For example, ex-prime minister Mahathir Mohamad frequently raised the specter of Jewish world domination as an argument for Muslim unity (e.g., in relation to the economic crisis of the late 1990s and the issue of Palestine), Malay translations of the *Protocols of the Elders of Zion* and *Mein Kampf* are widely available, and Holocaust denial is widespread even among academics (see Reid, 2010). Moreover, the loss of legitimacy of the ruling party post-Mahathir has made popular anti-Jewish racism as a means of diverting attention away from the state’s political failings and constructing an imagined enemy (Chirot and Reid, 1997; Reid, 2010; for a similar discussion in relation to the Middle East, see Gray, 2008).

It should be noted, however, that the label “Jew” is also frequently, though implicitly, applied to a range of broader enemies, including secularism, cosmopolitanism, globalization, Americanism, and capitalism and world banking (Bowen, 2006; Reid, 2010). As such, although directed explicitly at a community that does not exist in Malaysia, the Jewish conspiracy theory affords Malay-Muslim agents a means of implicitly scapegoating ethnic minorities and unifying Muslims around a political argument about threats to nationhood (see also Cohn-Sherbok, 2002). As has been discussed in relation to Syria, state-directed misinformation has the effect of strengthening popular nationalism, forcing opposition commentators to justify themselves through non-arguments, and reinforcing the state as a source of protection against an external threat (Wedeen, 1999). In short, it is quite possible that, in the context of Malaysia, the Jewish conspiracy theory fulfills context-specific needs among Malays.

The present study sought to investigate this possibility through two studies conducted among Malaysian Malays, the majority ethnic group in Malaysia. Study 1 conducted a preliminary examination of associations between belief in the Jewish conspiracy and variables related to anomie that have been included in previous studies. This study also examined Goertzel’s (1994) suggestion that conspiracy theories form part of a monological belief system. Study 2 extended the results of the first study by examining associations between belief in the Jewish conspiracy theory and a range of ideological variables. Although largely exploratory, the present work was driven by a motivation to examine the extent to which antecedents of conspiracist ideation identified in the West would remain stable in a non-Western setting.

## Study 1

Examining belief in the Jewish conspiracy theory is hampered by the lack of a suitable scale for the measurement of such a belief. As such, a first step of Study 1 was to construct an appropriate measure of belief in the Jewish conspiracy theory. A second step was the examination of associations between belief in the Jewish conspiracy theory and a range of individual difference factors that have been utilized in previous work (Goertzel, 1994; Abalakina-Paap et al., 1999; Swami et al., 2010, 2011). Specifically, Study 1 included measures of general conspiracist ideation (to test the hypothesis that conspiracy theories are monological in nature) and attitudes to authority, political cynicism, political alienation, self-esteem, and satisfaction with life (to test the suggestion that conspiracist ideation reflects feelings of anomie). Based on the above review of the literature, it was expected that belief in the Jewish conspiracy theory would be significantly associated with general conspiracist ideation and measures of anomie.

### Materials and methods

#### Ethical statement

The ethics committee at the Department of Psychology, University of Westminster, specifically approved this study. All participants provided written informed consent.

#### Participants

Participants of this study were 180 women and 188 men of Malay ancestry. Although Malaysia is ethnically heterogeneous, ethnic Malays make up a slight majority of the population (about 51%) and play a dominant role politically (by comparison, ethnic Chinese make up about 24% of the population). By constitutional definition, all Malays are Muslims who are required to adhere to Malay-Islamic customs. All participants were recruited from Kuala Lumpur, the capital and largest city in Malaysia. Participants ranged in age from 18 to 72 years (*M* = 44.15, SD = 12.90). In terms of educational qualifications, 40.8% had completed secondary schooling, 34.2% had a post-secondary diploma, 19.8% had an undergraduate degree, and 5.2% had a postgraduate degree.

#### Materials

##### Belief in the Jewish conspiracy theory

A novel scale was constructed to measure belief in the Jewish conspiracy theory. One item was adapted from Eysenck’s (1953) measure of opinions of the Jews and further items were constructed based on sources concerning the Jewish conspiracy theory in Southeast Asia (e.g., Chirot and Reid, 1997; Reid, 2010).Redundant items were excluded prior to data collection, leaving a final measure consisting of 12 items (see Table 1) that were rated on a seven-point Likert-type scale (1 = *strongly disagree*, 7 = *strongly agree*). The factor structure of the novel scale is reported in the Section “Results and Discussion” below.

**Table 1:** **Items and factor loadings of the novel measure of belief in the Jewish conspiracy theory from Study 1**.

Items	Factor 1	Factor 2
(1) Jews have too much power and influence in the world	0.90	0.17
(2) World banking is dominated by Jewish families	0.88	0.20
(3) Jews use their positions in world news media to foster a pro-Jewish agenda	0.81	0.34
(4) Jews have caused economic crises in this country for their own ends	0.76	0.22
(5) Jews are to blame for the detrimental effects of globalization in this country	0.74	0.12
(6) Jews are attempting to establish a secret world government	0.72	0.31
(7) Jews are secretly running the United States government in collaboration with Israel	0.69	0.12
(8) Jews are manipulating capitalism for their own ends	0.66	0.17
(9) The Holocaust is a myth fabricated to serve Jewish interests	0.58	0.18
(10) Jews are responsible for the social and moral ills in this country	0.52	0.87
(11) Jews are using political groups and organizations to destabilize this country	0.45	0.76
(12) Organizations such as the Freemasons are a means for Jews to establish a secret world government	0.39	0.69

*Items were rated on a seven-point Likert-type scale (1 = *strongly agree*, 7 = *strongly disagree*). Items were presented in a random order in the survey*.

##### Conspiracist ideation

The Belief in Conspiracy Theories Inventory (Swami et al., 2010, 2011) was used to measure general conspiracist ideation. This is a 15-item measure that describes a range of prominent and internationally recognizable conspiracy theories. (Sample item: “A powerful and secretive group, known as the New World Order, are planning to eventually rule the world through an autonomous world government, which would replace sovereign governments.”) The scale does not contain any items relating to Jewish conspiracy theories. All items were rated on a nine-point Likert-type scale (1 = *completely false*, 9 = *completely true*) and an overall score was computed as the mean of all items. Higher scores on this scale reflect greater belief in conspiracy theories. Previous work has shown that this scale is one-dimensional and has good internal consistency (Swami et al., 2010, 2011). In addition, the scores on the scale correlate strongly with scores from a non-event based, generic measure of conspiracist ideation (*r* = 0.88; Brotherton et al., 2012). In the present work, Cronbach’s α for this scale was 0.90.

##### Attitudes to authority

To measure attitudes to authority, this study used Reicher and Emler’s (1985) Attitudes to Authority Scale, which was adapted by Swami et al. (2010) to exclude items referring to fairness of school rules. The adapted measure consists of 10 items that relate to institutional authority, bias by those in authority, and the absolute priority of rules. (Sample item: “The main purpose of the law is to keep things as they are in a society that favors the rich.”) All items were rated on a five-point Likert-type scale (1 = *strongly disagree*, 5 = *strongly agree*) and an overall score was computed as the mean of all items following reverse-coding of some items. Higher scores on this scale reflect more negative attitudes to authority. This adaptation of the scale has been shown to have adequate internal consistency (Swami et al., 2010, 2011). Cronbach’s α for this measure was 0.72.

##### Political cynicism

The 13-item Political Cynicism Scale (Citrin and Elkins, 1975) was used to measure concern for public interest, idealism, and political determination. (Sample item: “Most politicians are really willing to be truthful to the voters.”) All items were rated on an *Agree*-*Disagree* format (1 = *agree*, 2 = *disagree*) and an overall score was computed as the sum of all items following reverse-coding of some items. Higher scores on this scale indicate greater political cynicism. The Political Cynicism Scale has good internal consistency and patterns of validity (Citrin and Elkins, 1975). Cronbach’s α for this measure was 0.78.

##### Political alienation

To measure political alienation, Malik’s (1982) Political Alienation Scale was used. This scale consists of five items (sample item: “Sometimes governmental and political affairs look so complex that I am unable to understand them”) that measure an individual’s sense of political efficacy and attitudes toward the political system. Items were rated on an *Agree-Disagree* format (1 = *disagree*, 2 = *agree*) and an overall score was computed as the sum of all items following reverse-coding of some items. Higher scores on this scale indicate greater political alienation. Malik (1982) reported that the scale has good internal consistency. In the present study, Cronbach’s α for this measure was 0.72.

##### Self-esteem

Self-esteem was measured using Rosenberg’s Self-Esteem Scale (Rosenberg, 1965; Malay translation: Swami, 2012). This is a 10-item measure that taps an individual’s self-worth (sample item: “On the whole, I am satisfied with myself”). All items were rated on a four-point Likert-type scale (1 = *strongly disagree*, 4 = *strongly agree*) and an overall score was computed by taking the mean of all items. Five items were reverse-coded prior to analysis and, for the Malay version, one item is allowed to load negatively (for a discussion, see Swami, 2012). Higher scores on this scale indicate higher self-esteem. The Malay version of this scale has been shown to have adequate internal consistency, good test-retest reliability after 5 weeks, and good patterns of validity. Cronbach’s α for this measure was 0.73.

##### Satisfaction with life

The Satisfaction with Life Scale (Diener et al., 1985; Malay translation: Swami and Chamorro-Premuzic, 2009) was used to measure subjective life satisfaction (sample item: “I am satisfied with my life”). The scale consists of five items that were rated on a five-point Likert-type scale (1 = *strongly disagree*, 5 = *strongly agree*). An overall score was computed as the mean of all five items and higher scores indicate greater satisfaction with life. The Malay version of this scale has been shown to be one-dimensional, with good internal consistency (Swami and Chamorro-Premuzic, 2009). In the present study, Cronbach’s α for this measure was 0.82.

##### Demographics

Participants provided their demographic details consisting of sex, age, religion, and highest educational qualifications.

#### Procedure

Unless otherwise stated above, all measures were initially prepared in English and were translated into Bahasa Malaysia (a standardized form of Malay) using the back-translation method (Breslin, 1970). The author initially prepared Malay translations of the scales, which were then back-translated into English by an independent translator unaffiliated with the study. Disputed phrasing was then settled by agreement between the author and the translator. All participants were recruited opportunistically from public locations in Kuala Lumpur by a research assistant. Potential participants were invited to take part in a study on political attitudes and, upon agreement to participate, provided informed consent. Participants completed a paper-and-pencil survey in a quiet location and returned the survey to the research assistant in a sealed envelope. The order of presentation of the above scales was randomized for each participant. All data were treated confidentially and anonymously, and participants were provided with a debrief sheet once they had returned their surveys. Participation was on a voluntary basis and participants were not remunerated for participation.

### Results and discussion

In order to examine the factor structure of the novel belief in the Jewish conspiracy theories scale, an exploratory factor analysis was computed using varimax rotation. The number of factors to be extracted was determined by factor eigenvalues (λ) above 1.0, the scree-plot criterion (Cattell, 1966), and an extraction criterion of 0.30 (Kline, 1986). The size of the Kaiser–Meyer–Olkin measure of sampling adequacy, KMO = 0.92, and Bartlett’s test of sphericity, χ^2^(66) = 3511.30, *p* < 0.001, indicated that the items had adequate common variance for factor analysis (Tabachnick and Fidell, 2007). A principal components analysis was, therefore, conducted on all 12 items of the scale.

The results of the factor analysis indicated the existence of two factors with λ > 1.0 after three iterations. However, the scree-plot showed a steep decline between the first (λ = 4.67) and second factors (λ = 2.89), and factor loadings indicated that all items loaded on to the dominant factor (see Table 1), which explained 38.8% of the variance. Based on these results and the criteria indicated above, the one-dimensional factor structure was preferred over a two-factor solution. An overall score of belief in the Jewish conspiracy theory was, therefore, calculated as the mean of all 12 items. Cronbach’s α for this overall factor was very good (0.94). An independent-samples *t*-test showed that there was no significant difference between women and men on this measure, *t*(366) = 0.41, *p* = 0.682, *d* = 0.04. Descriptive statistics for this measure (reported in Table 2) indicated that participants’ mean responses were around the mid-point of the scale.

**Table 2:** **Associations between belief in the Jewish conspiracy theory and all other variables included in Study 1**.

	(1)	(2)	(3)	(4)	(5)	(6)	(7)	(8)
(1) Belief in the Jewish conspiracy theory		0.22**	0.07	0.09	0.13*	−0.02	0.05	0.18**
(2) General conspiracist ideation			0.17*	0.20**	0.06	−0.03	−0.03	−0.14*
(3) Attitudes to authority				0.36**	0.11*	0.07	0.10	0.13*
(4) Political cynicism					0.40**	−0.02	−0.03	−0.11*
(5) Political alienation						0.09	0.10	−0.22**
(6) Self-esteem							0.50**	0.23**
(7) Satisfaction with life								0.19**
(8) Participant age								
*M*	3.70	4.13	3.25	17.35	7.02	2.08	3.10	44.15
SD	1.91	1.40	0.50	3.50	1.44	0.48	0.76	12.90

*N* = 368. *p < 0.05, **p < 0.001.

To examine antecedents of belief in the Jewish conspiracy theory, bivariate correlations were conducted between overall scores as derived above and all remaining variables (general conspiracist ideation, attitudes to authority, political cynicism, political alienation, self-esteem, satisfaction with life, and participant age). As can be seen in Table 2, belief in the Jewish conspiracy theory was significantly and positively correlated with general conspiracist ideation, although the strength of the correlation was weak. Belief in the Jewish conspiracy theory was also positively correlated with participant age and political cynicism, although the strength of these correlations was likewise weak. Belief in general conspiracy theories was positively correlated with attitudes to authority and political cynicism, and negatively correlated with participant age.

To examine the predictive strength of these associations, a multiple linear regression was conducted with belief in the Jewish conspiracy theory as the criterion variable and all remaining factors as predictor variables. The overall regression was significant, *F*(7, 367) = 9.69, *p* < 0.001, Adj. *R*^2^ = 0.11, but the only significant predictor was general conspiracist ideation (*B* = 0.08, SE = 0.04, β = 0.12, *t* = 2.58, *p* = 0.010). When the regression analysis was repeated with general conspiracist ideation as the criterion variable, the regression was again significant, *F*(6, 367) = 4.11, *p* = 0.001. The only significant predictors in this model were political cynicism (*B* = 0.07, SE = 0.02, β = 0.17, *t* = 2.86, *p* = 0.004) and participant age (*B* =  −0.01, SE = 0.01, β = −0.12, *t* = −2.25, *p* = 0.027).

Overall, the results of Study 1 provided only limited support for the hypotheses. Although belief in the Jewish conspiracy theory was significantly associated with general conspiracist ideation, which supports the idea of a monological belief system, the association was relatively weak. Furthermore, other measures of anomie did not emerge as significant predictors of belief in the Jewish conspiracy theory, which stands in contrast to the findings of previous work. In terms of general belief in conspiracy theories, the results indicated that politically cynical individuals were more likely to hold stronger conspiracist beliefs. Again, however, the association was weak and further variables associated with anomie did not emerge as significant predictors. In short, the results of Study 1 appear to suggest that measures of anomie are not strong predictors of conspiracist ideation among Malaysian Malays.

## Study 2

It is possible that the results of Study 1 reflect the fact that belief in the Jewish conspiracy theory among Malaysian Malays stems, not from feelings of powerlessness but rather from implicit resentment toward ethnic minorities (particularly the Chinese), as well explicit hostility toward Western secularism and cosmopolitanism (Hadler, 2004). That is, rather than being monological in the sense proposed by Goertzel (1994), belief in the Jewish conspiracy theory may serve a specific ideological purpose within the Malaysian geopolitical context. If this is the case, then the lack of significant associations between belief in this conspiracy theory and measures of anomie is unsurprising, as the conspiracy theory is not driven by traditional feelings of powerlessness, disadvantage, or voicelessness (in fact, among some Malaysian Malays, it may be just the opposite).

Study 2, therefore, sought to extend the findings of the first study by examining the relationships between belief in the Jewish conspiracy theory and a range of ideological variables. Specifically, this study included measures of right-wing authoritarianism (a measure of support for traditional social norms and submission to authority), social dominance orientation (a measure of preference for hierarchical social systems), anti-Israeli attitudes (which has been argued to be a socially acceptable cover for hostility toward Jews; Cohen et al., 2009), and a measure of modern racism (adapted to refer to the Chinese). In addition, participants also completed a measure of general conspiracist ideation, as in Study 1, which allowed for a test of the relative strength of these variables in predicting belief in the Jewish conspiracy theory.

### Materials and methods

#### Ethical statement

The ethics committee at the Department of Psychology, University of Westminster, specifically approved this study. All participants provided written informed consent.

#### Participants

Participants of Study 2 were 162 women and 152 men of Malay ancestry, recruited from Kuala Lumpur. Participants ranged in age from 18 to 68 years (*M* = 40.89, SD = 13.04) and all were Muslims. In terms of educational qualifications, 38.8% had completed secondary education, 36.3% had a post-secondary diploma, 19.4% had an undergraduate degree, and 5.4% had a postgraduate degree.

#### Materials

##### Belief in the Jewish conspiracy theory

Participants completed the 12-item measure of belief in the Jewish conspiracy theory, as described in Study 1. Based on the above results, an overall score for this scale was computed as the mean of all 12 items. Cronbach’s α for this scale in Study 2 was 0.90.

##### General conspiracist ideation

Participants completed the Belief in Conspiracy Theories Inventory (Swami et al., 2010, 2011), as described in Study 1. Cronbach’s α for this measure was 0.88.

##### Right-wing authoritarianism

To measure authoritarianism, the short form of the Right-Wing Authoritarianism Scale (Zakrisson, 2005) was used. This is a 15-item short version of the original 30-item measure (Altemeyer, 1988) and measures the degree to which an individual supports traditional social norms and submission to authority. (Sample item: “Our country needs a powerful leader, in order to destroy the radical and immoral currents prevailing in society today.”) All items were rated on a seven-point Likert-type scale (1 = *strongly disagree*, 7 = *strongly agree*) and an overall score was computed as the mean of all 15 items. Higher scores on this scale provide an index of greater right-wing authoritarianism. The short form of this scale has good psychometric properties (Zakrisson, 2005). In the present study, Cronbach’s α for this measure was 0.86.

##### Social dominance orientation

The 16-item Social Dominance Orientation Scale (Sidanius and Pratto, 1999) was used to measure an individual’s preference for hierarchical social systems and desire for one’s social group to dominate over others. (Sample item: “To get ahead in life, it is sometimes necessary to step on other groups.”) All items were rated on a seven-point Likert-type scale (1 = *strongly disagree*, 7 = *strongly agree*) and an overall score was computed as the mean of all 16 items. Higher scores on this scale indicate greater social dominance orientation. Sidanius and Pratto (1999) provide evidence of the scale’s psychometric properties. In this study, Cronbach’s α for this measure was 0.89.

##### Attitudes toward Israel

Cohen et al. (2009) devised the Attitudes toward Israel Scale, a 10-item measure, to measure an individual’s pro-Israeli sentiment. (Sample item: “I strongly support the Israeli cause.”) All items were rated on a five-point Likert-type scale (1 = *strongly disagree*, 5 = *strongly agree*) and an overall score was computed as the mean of all items. Higher scores on this scale reflect more pro-Israeli attitudes. Cohen et al. (2009) reported that the measure has good internal consistency, which was also the case in the present study (Cronbach’s α = 0.92).

##### Modern racism

This study included the Modern Racism Scale (McConahay et al., 1981; McConahay, 1983, 1986), which measures an individual’s degree of ethnic prejudice. The scale has been successfully adapted to different target groups (e.g., Echebarria-Echabe and Guede, 2007) and in the present study, the six-item measure was adapted to refer to the Chinese. (Sample item: “Over the past few years, the government and news media have shown more respect for the Chinese than they deserve.”) All items were rated on a five-point Likert-type scale (1 = *strongly disagree*, 5 = *strongly agree*) and an overall score was computed as the mean of all items. Higher scores on this scale reflect greater prejudice toward the Chinese. The original version of the scale has been shown to have good psychometric properties (McConahay et al., 1981). In the present study, Cronbach’s α for this measure was 0.82.

##### Demographics

Participants provided their demographic details consisting of sex, age, religion, and highest educational qualifications.

#### Procedure

The procedures of Study 2 were identical to those of Study 1, with the exception that participation was limited to those who had not taken part in Study 1.

### Results and discussion

Descriptive statistics for, and bivariate correlations between, all variables included in Study 2 are reported in Table 3. As can be seen, stronger belief in the Jewish conspiracy theory was significantly associated with more anti-Israeli attitudes, higher racism, greater social dominance orientation, greater right-wing authoritarianism, stronger belief in general conspiracy theories, and older age. The correlations were strongest for attitudes toward Israel and modern racism and weakest for general conspiracist ideation. Belief in general conspiracy theories was significantly and negatively correlated with right-wing authoritarianism, social dominance orientation, and participant age (*R*s = −0.14 to −0.20), but none of the other variables.

**Table 3:** **Associations between belief in the Jewish conspiracy theory and all other variables included in Study 2**.

	(1)	(2)	(3)	(4)	(5)	(6)	(7)
(1) Belief in the Jewish conspiracy theory		0.17*	0.33**	0.37**	−0.52**	0.50**	0.17*
(2) General conspiracist ideation			−0.20**	−0.22**	−0.06	0.02	−0.14*
(3) Right-wing authoritarianism				0.36**	−0.26*	0.50**	0.19*
(4) Social dominance orientation					−0.33**	0.47**	0.22**
(5) Attitudes toward Israel						−0.27**	0.07
(6) Modern racism							0.19**
(7) Age							
*M*	3.77	4.01	3.78	3.99	1.12	3.92	40.89
SD	1.91	1.38	0.46	1.20	0.50	0.87	13.04

*p < 0.05, **p < 0.001.

To examine the predictive strength of these associations, a multiple linear regression was conducted with belief in the Jewish conspiracy theory as the criterion variable and all remaining factors as predictor variables. The overall regression was significant, *F*(6, 313) = 34.17, *p* < 0.001, Adj. *R*^2^ = 0.39. Of the variables entered into the model, the strongest predictor was attitudes toward Israel (*B* = −0.68, SE = 0.06, β = −0.56, *t* = −12.14, *p* < 0.001). Other variables that emerged as significant predictors were modern racism (*B* = 0.07, SE = 0.01, β = 0.17, *t* = 4.76, *p* < 0.001), right-wing authoritarianism (*B* = 0.02, SE = 0.01, β = 0.15, *t* = 3.12, *p* = 0.002), and social dominance orientation (*B* = 0.02, SE = 0.01, β = 0.14, *t* = 3.00, *p* = 0.006). General conspiracist ideation and participant age did not emerge as significant predictors in this analysis.

The results of Study 2 indicate that belief in the Jewish conspiracy theory among Malaysian Malays was significantly associated with anti-Israeli attitudes, modern racism directed at Chinese, right-wing authoritarianism, and social dominance orientation. When these ideological variables had been accounted for, general conspiracist ideation did not emerge as a significant predictor. Overall, these results suggest that belief in the Jewish conspiracy theory may serve particular ideological needs rather than conform to a monological belief system, at least among the present sample. That is, the Jewish conspiracy theory may not be viewed in the same way as other conspiracy theories, but rather appears to reflect specific political expressions within a particular geopolitical context.

## General Discussion

The results of the present studies suggest that, in the Malaysian context, belief in the Jewish conspiracy theory may only be weakly associated with belief in other conspiracy theories. Rather, belief in this conspiracist, anti-Semitic narrative appears to serve ideological demands and needs that may be more pronounced in the Malaysian context. For example, various authors have noted the upsurge of anti-Jewish rhetoric among politically conservative Malaysians, motivated in part by the question of Palestine, but also broader concerns about the threat from secularism, cosmopolitanism, globalization, and capitalism. In this context, the Jewish conspiracy theory should be viewed through the lens of the particular geopolitical context in Malaysia, especially in relation to the resurgence of political Islam and Muslim victimhood (Reid, 2010).

Interestingly, the results of Study 2 also indicate that belief in the Jewish conspiracy theory was associated with prejudice toward the Chinese. Several scholars have suggested that, in Malaysia, anti-Jewish rhetoric may represent an implicit attack on the Chinese, particularly Chinese commerce and business elites (Hadler, 2004). Because explicitly targeting the Chinese is both illegal and politically unwise, the Jewish conspiracy theory may offer a means of expressing an anti-Chinese polemic that would otherwise remain suppressed (Reid, 2010). That is, to the extent that explicit anti-Chinese rhetoric must remain hidden, the anti-Jewish conspiracy theory provides a crude tool for the expression of racist sentiment. In a political climate marked by the exploitation of race for political ends, but in which explicitly anti-Chinese rhetoric must remain hidden, conspiracy theories about the Jews may serve particular ideological needs (Hadler, 2004; Reid, 2010).

It is also interesting to note that, in Study 1, belief in general conspiracy theories was significantly predicted by political cynicism, but not other measures of anomie. Moreover, Study 2 showed that belief in general conspiracy theories was significantly associated with right-wing authoritarianism and social dominance orientation, albeit weakly. These results stands in contrast to previous findings among Western samples, which indicate that belief in conspiracy theories is significantly associated with measures of anomie (Goertzel, 1994; Abalakina-Paap et al., 1999; Swami et al., 2010, 2011). Additionally, the negative association between belief in general conspiracy theories and right-wing authoritarianism appears to be stronger in the present study than has been reported among central European participants (Swami et al., 2012a). In short, the present results provide preliminary support for the suggestion that conspiracist ideation may play different roles in different cultural contexts.

As Gray (2008, p. 167) has discussed, “There is no single theory of conspiracism that simply and neatly can explain conspiracism, much less one that can be taken from the Western experience and superimposed onto [other cultures] to explain both the breadth and frequency of conspiracy discourse.” Rather, when seeking to evaluate belief in conspiracy theories in specific national contexts, it will be important for scholars to carefully consider the uniqueness of the local political, economic, and cultural climate. Certainly, the present study suggests that explanations related to anomie and powerlessness may be insufficient to explain the maintenance and spread of conspiracy theories in Malaysia. Of course, this is not to suggest that the study of conspiracism in the West will not be beneficial for understanding it in other cultural contexts. Rather, accumulating evidence suggests that there may be limits to the applicability and transferability of the former onto the latter (Gray, 2008).

Nevertheless, this discussion of cultural specificity should be interpreted with some caution. For one thing, previous studies of conspiracist ideation among Western samples have tended to focus on conspiracy theories that tend to be stigmatized or outside the mainstream, which contrasts with the ubiquity of the Jewish conspiracy theory among Malaysian Malays. As such, it is quite possible that more mainstream conspiracy theories in the West will also show similar patterns of antecedents as the Jewish conspiracy theory in Malaysia. In short, more work is needed to ascertain the extent to which there is a cultural gap in the socio-political functions of conspiracism in the West. In a similar vein, it would also be useful to further explore whether, or the extent to which, the relationship between the Jewish conspiracy theory and ant-Chinese sentiment among Malaysian Malays in fact reflects a more generalized reaction that views any outsider as having sinister motives.

This study is also not without other limitations. Socially desirable responding is a real concern, particularly as participants may not have wished to disclose politically or socially sensitive information (despite the steps taken, such as assurances of anonymity). In addition, the opportunistic method of sampling means that the present results may not be generalizable to the wider Malay public. Indeed, and conversely, the present set of results may represent phenomena that are specific to Malaysian Malays, and future studies would do well to replicate these results in other cultural settings. For example, it is unclear whether the same pattern of results would be found in Western settings where anti-Semitic sentiments are more culturally circumscribed than in Malaysia. Furthermore, although steps were taken to ensure that the scales used in the present study were appropriately back-translated, it is possible that translational issues affected the present set of findings. Finally, future studies could include further variables that were neglected in the present work, such as religiosity (which may be particularly important given the issue of Muslim victimhood and its relevance to belief in the Jewish conspiracy theory).

Even so, the present results raise important questions about the nature and functions of anti-Jewish conspiracy theories in the Malaysian context. It would appear that, among Malaysian Malays at least, the Jewish conspiracy theory serves specific ideological functions that are distinct from other conspiracy theories. More broadly, the present findings suggest that anti-Jewish rhetoric among Malaysian Malays should not be dismissed as a marginal phenomenon. Rather, it would appear that, as the Jewish conspiracy is espoused by politicians and the mainstream media, boundaries between acceptable and unacceptable political discourse becomes blurred (for a similar discussion concerning Yugoslavia, see Byford and Billig, 2001). In this context, there is an urgent need to more carefully locate the ideological functions served by such conspiracy theories and to begin the difficult task of combating anti-Jewish and other racist rhetoric.

## Conflict of Interest Statement

The author declares that the research was conducted in the absence of any commercial or financial relationships that could be construed as a potential conflict of interest.
